# Concurrent X chromosome inactivation and upregulation during non-human primate preimplantation development revealed by single-cell RNA-sequencing

**DOI:** 10.1038/s41598-021-89175-7

**Published:** 2021-05-05

**Authors:** Ana Luíza Cidral, Joana C. Moreira de Mello, Joost Gribnau, Lygia V. Pereira

**Affiliations:** 1 National Laboratory for Embryonic Stem Cells (LaNCE), Department of Genetics and Evolutionary Biology, Institute of Biosciences, University of São Paulo, São Paulo, SP 05508-090 Brazil; 2 Department of Developmental Biology, Oncode Institute, Erasmus MC University Medical Center, 3015GE Rotterdam, The Netherlands

**Keywords:** Development, Epigenetics

## Abstract

In mammals, dosage compensation of X-linked gene expression between males and females is achieved by inactivation of a single X chromosome in females, while upregulation of the single active X in males and females leads to X:autosome dosage balance. Studies in human embryos revealed that random X chromosome inactivation starts at the preimplantation stage and is not complete by day 12 of development. Alternatively, others proposed that dosage compensation in human preimplantation embryos is achieved by dampening expression from the two X chromosomes in females. Here, we characterize X-linked dosage compensation in another primate, the marmoset (*Callithrix jacchus*). Analyzing scRNA-seq data from preimplantation embryos, we detected upregulation of *XIST* at the morula stage, where female embryos presented a significantly higher expression of *XIST* than males. Moreover, we show an increase of X-linked monoallelically expressed genes in female embryos between the morula and late blastocyst stages, indicative of XCI. Nevertheless, dosage compensation was not achieved by the late blastocyst stage. Finally, we show that X:autosome dosage compensation is achieved at the 8-cell stage, and demonstrate that X chromosome dampening in females does not take place in the marmoset. Our work contributes to the elucidation of primate X-linked dosage compensation.

## Introduction

In mammals, dosage compensation between the single X chromosome in males and the two X in females is achieved by the process of X chromosome inactivation (XCI) which leads to the transcriptional silencing of one X in females^1^. In eutherian mammals the process is initiated during preimplantation development with the expression of the long non-coding RNA *XIST* from the future inactive X (Xi)^2,3^. Until recently, most studies of XCI initiation were performed in mice due to the ease in performing experiments in this animal model and the capability of mouse embryonic stem cells (mESCs) to undergo XCI when differentiated in vitro. Thus, in mice, XCI is initiated at the 4-cell stage with *XIST* being expressed exclusively from the paternal allele, leading to imprinted XCI (reviewed in^4^). At the blastocyst stage, cells from the epiblast (EPI) reactivate the inactive paternal X and, upon implantation, undergo a second round of XCI where each cell randomly inactivates either the paternal or the maternal X.

In contrast, studies of XCI initiation in humans have been hampered by difficulties in performing experiments in human embryos and lack of an adequate in vitro model system, since human embryonic stem cells (hESCs) are mostly in a post-XCI state^5^. Nevertheless, several differences between the process in mice and humans have been observed, including initiation of *XIST* expression at the 8-cell stage^6^, *XIST* expression from both X chromosomes in female or from the single X chromosome in male embryos^7,8^ and lack of imprinted XCI^9^.

Intriguingly, using single-cell RNA sequencing (scRNA-seq) data from human preimplantation embryos, Petropoulos et al*.*^8^ identified X-linked dosage compensation in female blastocysts in the absence of XCI. The authors proposed a dampening process of X-linked gene expression from both active X chromosomes as an alternative mechanism of dosage compensation in human embryos^8^. Using a more stringent analysis combining the aforementioned and additional scRNA-seq data, Moreira de Mello et al*.* excluded the hypothesis of X-chromosome dampening and showed evidence that initiation of random XCI takes place between the morula and early blastocyst stage in humans^10^. More recently, Zhou et al*.* showed that XCI initiates around the time of implantation and is not complete by day 12 of human development^11^. The conflicting results regarding *XIST* expression and timing of initiation of XCI pose a challenge in the efforts to uncover the mechanisms of dosage compensation in humans.

In this study, we aimed to investigate features of the XCI in a species that is evolutionarily closer to humans than mice. The marmoset (*Callithrix jacchus*) is a new world monkey widely used in biomedical research, whose evolutionary distance from humans is around 40 million years. We analyzed scRNA-seq data from 14 marmoset embryos, from zygote to the late blastocyst stage^12^. Using allele-specific expression analysis we show that there is no evidence of X dampening during this primate’s early development and that random XCI is also initiated at the preimplantation stage, concomitant with upregulation of the active X in male and female embryos.

## Results

### Embryo sexing

The dataset originally contained the transcriptome of 196 cells obtained from 14 marmoset preimplantation embryos, from the zygote to the late blastocyst stage. After filtering out cells with low-quality RNAseq data, we retained 124 cells representing all the above-mentioned stages (Table 1).

**Table 1:** Summary of embryos and cells analyzed.

Embryo	Number of cells	Number of cells after filtering	Cell type
Zygote_1	1	1	–
Zygote_2	1	1	–
Zygote_3	1	-	–
4cell_1	4	4	Blastomere
8cell_1	7	5	Blastomere
8cell_2	8	5	Blastomere
CompactedMorula_1^a^	**22**	**15**	Morula
CompactedMorula_2	10	9	Morula
CompactedMorula_3^a^	**21**	**21**	Morula
EarlyICM_1	9	7	Inner cell mass
EarlyICM_2^a^	**10**	**8**	Inner cell mass
EarlyICM_3^a^	**23**	**8**	Inner cell mass
LateICM_2	39	26	Primitive endoderm, epiblast
LateICM_4^a^	**40**	14	Primitive endoderm, epiblast

^a^Female embryos.

Embryos at the same stage were compared in terms of the number of Y-linked expressed genes and their respective expression levels. This strategy is able to sex embryos after complete embryonic genome activation, at the 8-cell stage^12–14^. We identified two morulas, two early- and one late-blastocyst as female; and two 8-cell embryos, one morula, one early- and one late-blastocyst as male (Supplementary Fig. S1 and Table 1).

### Allele-specific expression

In order to analyze allele-specific expression, we identified heterozygous SNP positions in each embryo. For a SNP position to be considered, it had to be covered by at least 20 reads; and if a gene contained variants indicating mono and biallelic expression in the same cell, that gene was discarded from the analysis. In total, 16,311 heterozygous positions and 6985 informative genes were detected, of which 181 were located on the X chromosome.

Twenty three X-linked genes biallelically expressed were detected in male embryos (Supplementary Fig. S2). This is expected in cells from stages prior to embryonic genome activation (EGA) as maternal mRNAs are still present^12–14^. In later staged male embryos, X-linked biallelic expression may be detected due to misalignment of reads from genes located in the pseudoautosomal regions (PAR) or from genes with homologues in the Y and the X chromosomes^15^. In order to avoid interference from these genes, and since the marmoset PAR is not well described, we removed from the analysis all genes homologous to those in the human PAR and to those known to escape XCI (*escapees*), and all X-linked genes identified as biallelically expressed in at least one cell from a male embryo after the 8-cell stage (Supplementary Figs. S2 and S3). All subsequent analyses were performed using 140 informative X-linked genes resulting from this filtering step.

### *XIST* expression

**Figure 1:**
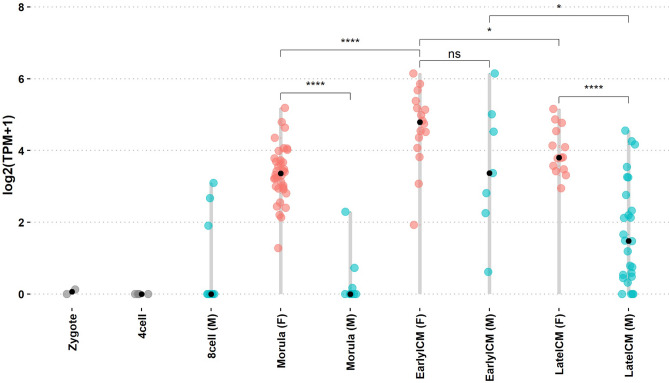
*XIST* expression in single cells during marmoset embryonic development. Each dot represents a single cell. Cells are grouped by embryonic stage and sex. Pink, female embryos (F); blue, male embryos (M). Distributions of *XIST* expression levels were compared using an unpaired Wilcoxon test. (*) P-value ≤ 0.05; (****) P-value ≤ 0.0001; (ns) not significant (P-value ≥ 0.05).

Induction of *XIST* expression is a hallmark of XCI initiation. In the marmoset embryos, *XIST* expression was first detected at the 8-cell stage in males (Fig. 1). *XIST* expression in female compacted morulas was significantly higher than in males at the same stage (P-value = 6.7 × 10^–6^). Surprisingly, at the early blastocyst stage, cells from the ICM from both male and female embryos expressed high levels of *XIST*. Although *XIST* expression persisted in the ICM of male late blastocyst, it was significantly downregulated (P-value = 4.1 × 10^–5^).

### Allele-specific X-linked gene expression

**Figure 2:**
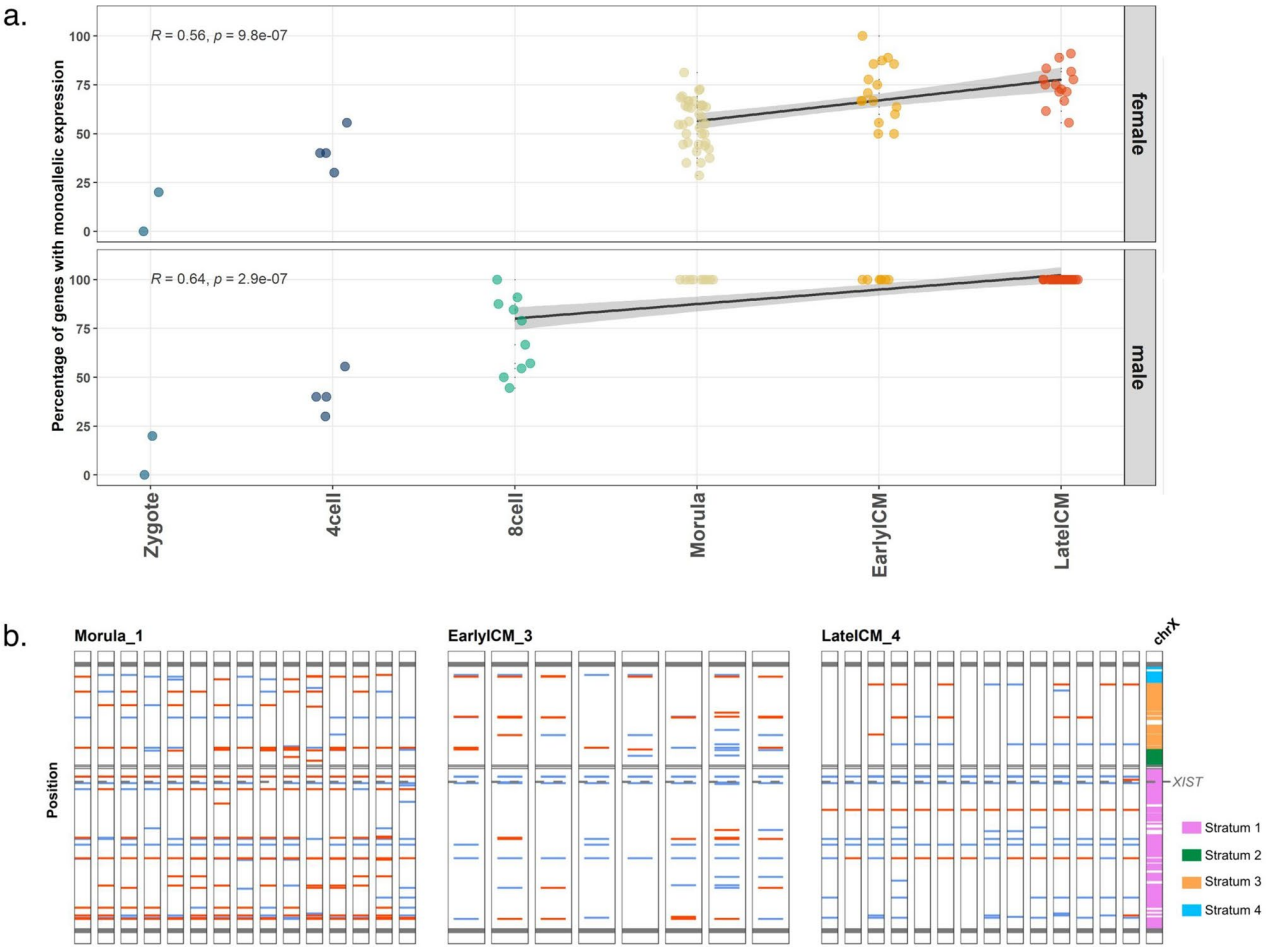
Allelic expression of X-linked genes. (**a**) Percentage of monoallelically expressed X-linked genes for each cell according to embryonic stage. Top panel: female embryos; bottom panel: male embryos. Each dot represents a single cell. Pearson’s r values (R) are depicted in each panel. Gray area indicates a 95% confidence interval. (**b**) Expression patterns of genes along the X-chromosome of female embryos. The color of each position is assigned according to the ratio of allelic expression based on the detected SNP; blue corresponds to monoallelic expression, while red indicates biallelic expression. Each column corresponds to a single cell. The dark grey lines indicate the telomeres; the light gray lines the centromere; and the dashed line corresponds to the putative marmoset *XIST locus*. ChrX: scheme of the X chromosome showing the evolutionary strata S1, S2, S3 and S4 based on human data^16,17^.

X chromosome inactivation leads to monoallelic expression of most X-linked genes in females. Thus, in order to further investigate the process during marmoset preimplantation embryo development, we calculated the proportion of monoallelically expressed genes in each embryonic stage (Fig. 2a). As expected, male embryos showed an increase of X-linked monoallelic expression between the 8-cell and late blastocyst stages, reflecting the degradation of maternal mRNA (Fig. 2a and Supplementary Fig. S4). In female embryos, we found a significant increase of monoallelic X-linked expression between the morula and blastocyst stages (Pearson’s r = 0.56; P-value = 9.8 × 10^–7^, Fig. 2a; and Supplementary Fig. S4), indicative of an ongoing XCI process. The same analysis performed in chromosome 10 did not reveal any significant increase of monoallelically expressed genes in males (Pearson’s r = 0.16: P-value = 0.27) and a small correlation in females (Pearson’s r = 0.26; P-value = 0.038) (Supplementary Fig. S5).

The location of all X-linked informative SNPs and their allelic-expression patterns in female embryos are shown in Fig. 2b and Supplementary Fig. S4. It is possible to see a cluster of genes close to the *XIST locus* with monoallelic expression in most of the cells at the early and late blastocyst stages.

Interestingly, we detected monoallelic expression of *XIST* from the alternative allele in one cell of the female early blastocyst. In another cell of the same embryo, the reference allele was monoallelically expressed, although with coverage of 14 reads, below our established threshold of 20 covering reads (Supplementary Fig. S6). Nevertheless, the detection of different *XIST* alleles being monoallelically expressed in different cells of the same embryo indicates that random XCI is taking place. In the same early blastocyst embryo where *XIST* showed monoallelic expression, we also detected three other genes located in a 1 Mb window from *XIST* being monoallelically expressed: *RNF12* and two uncharacterized *loci* (LOC100408393 and LOC100404148). In mice, *Rnf12* is an activator of XCI, participating in the upregulation of *XIST*^18–20^. During the *Xist* accumulation process, *Rnf12* is expressed exclusively from the future active X, and is downregulated very quickly after XCI is initiated^21^. Therefore, detection of monoallelic expression of *RNF12* further suggests ongoing XCI in the marmoset early blastocyst.

### X chromosome dampening

**Figure 3:**
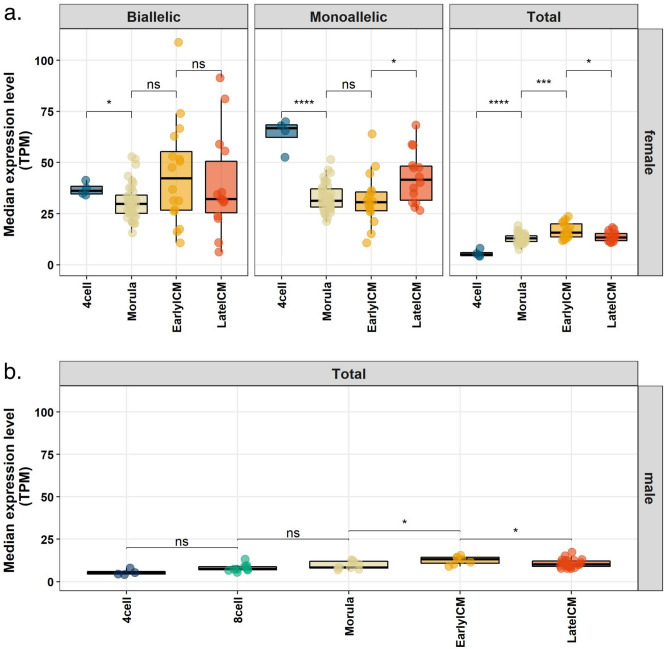
Expression levels of X-linked genes in marmoset preimplantation embryos. Each dot represents a single cell. Distribution of the median expression levels of X-linked genes as follows: (**a**) left panel: biallelically expressed; middle panel: monoallelically expressed; right panel: all X-linked expressed genes in each cell of female embryos (biallelic, monoallelic and non-informative). (**b**) Median expression levels of all expressed X-linked genes in each cell of male embryos. Stages were compared to each other using an unpaired Wilcoxon test. (*) P-value ≤ 0.05; (***) P-value ≤ 0.001; (****) P-value ≤ 0.0001; (ns) not significant (P-value ≥ 0.05).

According to Petropoulos et al., dampening leads to dosage compensation in human preimplantation embryos by decreased expression of biallelically expressed X-linked genes^8^. In order to test the occurrence of X chromosome dampening in the marmoset, we calculated the median expression levels of biallelically expressed X-linked genes in female embryos during development (Fig. 3; Supplementary Fig. S7). If dampening of the two active X chromosomes occurred, we should detect a decrease in the median expression level of those genes during development. We found no significant differences in the expression levels of biallelically expressed X-linked genes among morula, early and late blastocyst stages (Fig. 3a), refuting the occurrence of X chromosome dampening.

Together, these data show that, while there is a growing number of monoallelically expressed genes indicative of ongoing XCI, genes that are still transcribed from both X chromosomes are not downregulated (dampened) between the morula, early and late blastocyst stages. Interestingly, we found a significant increase of the median expression levels of monoallelically expressed X-linked genes between the early and late blastocyst stages in females (Fig. 3b).

### X chromosome upregulation

Upregulation of the single active X in mammalian cells was proposed as a mechanism of dosage compensation between that chromosome and the pairs of autosomes^22^. Indeed, X upregulation has been observed in humans, marsupials and mice^10,23,24^. In order to further investigate the increase in X-linked gene expression during marmoset embryonic development, we looked at the expression levels of all X-linked genes in both male and female embryos. We identified an increase in total X-linked median expression levels between morula and early blastocyst stage in both males and females consistent with upregulation of the X chromosome (Fig. 3a,b; P-value = 3.6 × 10^–4^ and 0.031 for females and males, respectively).

**Figure 4:**
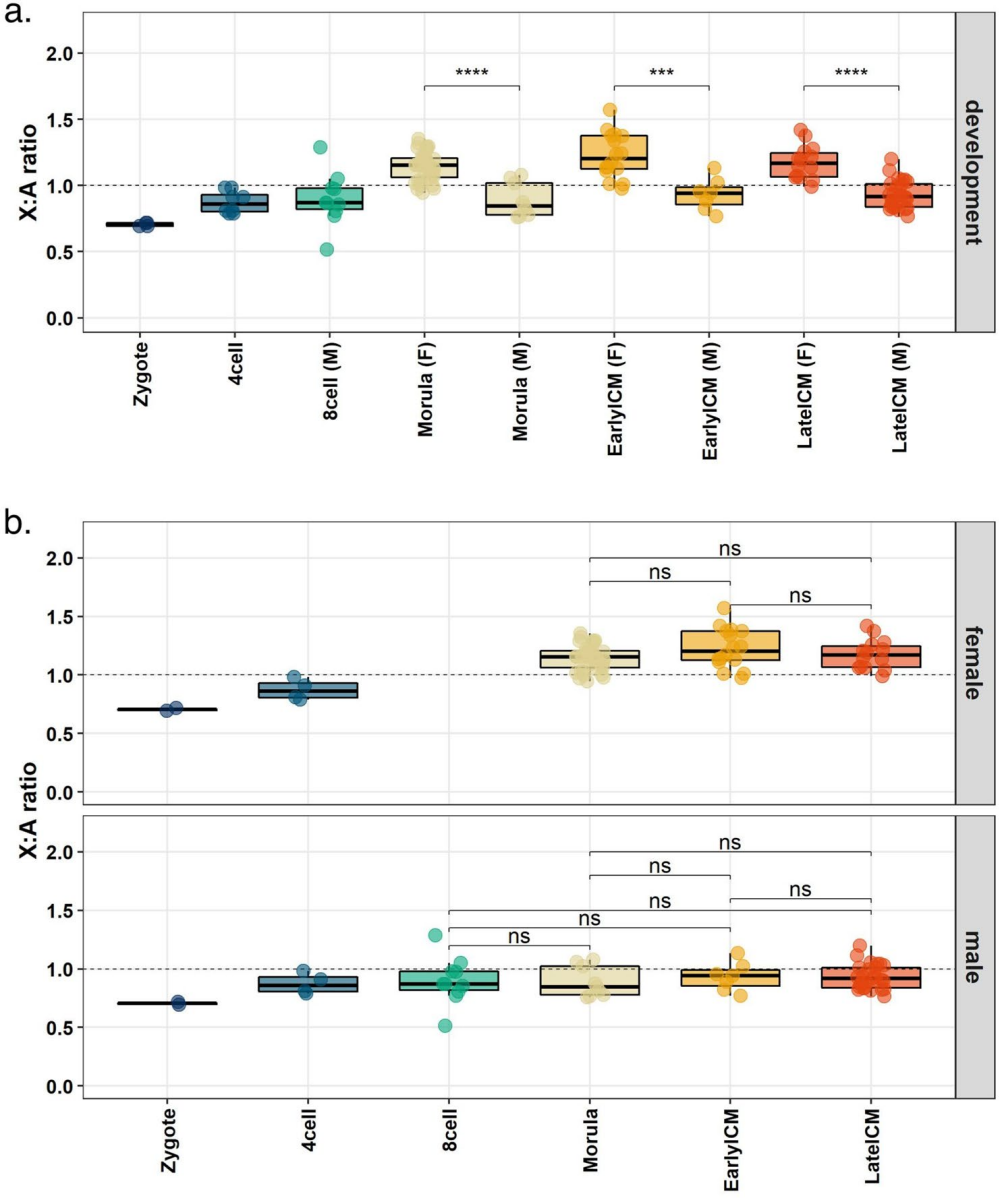
X-to-autosome dosage compensation. (**a**) X:A ratio distribution calculated per cell of each embryonic stage during marmoset preimplantation development. Unpaired Wilcoxon tests were used to compare males and females at the same developmental stage. (***) P-value ≤ 0.001; (****) P-value ≤ 0.0001. (**b**) Same as in (**a**) with one panel for each sex. Top panel: female embryos; bottom panel: male embryos. Note that, although not sexed, zygotes and 4-cell stage embryos are duplicated in order to maintain panels uniform. Unpaired Wilcoxon tests were used to compare different stages in males and females separately. (ns) not significant (P-value ≥ 0.05).

In order to test if upregulation grants the X chromosome an expression level similar to the autosomes, we calculated the X:A ratio for each cell grouped by embryonic stage and sex (Fig. 4; Supplementary Fig. S8). Our results indicate that marmoset male embryos achieve dosage compensation between X chromosome and autosomes earlier than humans: at the 8-cell stage the X:A ratio is already close to 1 (Fig. 4b), whereas in humans this is not yet achieved by the blastocyst stage^10^. In marmoset female morulas, early and late blastocyst, the X:A ratio was greater than 1 but less than 2 (Fig. 4b), consistent with the occurrence of both X upregulation and incomplete XCI as observed in human embryos^10^. Moreover, X:A ratios were significantly different between male and female in all analyzed stages (Fig. 4a; morula, P-value = 1.9 × 10^–5^; early blastocyst, P-value = 5.4 × 10^–5^; late blastocyst P-value = 4.8 × 10^–7^), further indicating incomplete XCI. Together with the analysis of allele-specific expression level (Fig. 3), these results suggest that X upregulation takes place and is complete earlier than XCI during marmoset preimplantation embryonic development.

## Discussion

Despite remarkable advances in dissecting the mechanisms of XCI in mice, much less is known about the process during human embryonic development. Still, important differences between the process in the two species have been identified, including lack of imprinted XCI in humans^9^ and different timing of random XCI initiation^6–8,10^, although there is still disagreement regarding the latter. While some studies have identified initiation of random XCI in pre and perimplantation embryo stages^5,10,11^, others argue that dosage compensation in human blastocysts is achieved by X chromosome dampening^8,25^. Although the hypothesis of dampening has been also refuted in pigs^26^, the study of X-linked dosage compensation in an animal model evolutionarily closer to humans may contribute in elucidating these issues. Therefore, here we sought to characterize the dynamics of X chromosome regulation during preimplantation development in another primate, the marmoset (*Callithrix jacchus*).

By analysing scRNA-seq data, we were able to perform a chromosome-wide analysis of X-linked genes in marmoset preimplantation embryos. Our results are consistent with an ongoing process of XCI in the early blastocyst of the female marmoset, characterized by an increase in *XIST* expression and in the percentage of monoallelically expressed X-linked genes, as observed in human preimplantation embryos^10,11^.

Monoallelic expression of alternative *XIST* alleles in different cells of the same early blastocyst embryo suggests that XCI takes place in a random pattern. This is expected since the expression data were of cells from the ICM and adult marmoset females present random XCI^27^. In mice, extraembryonic tissues present imprinted inactivation of the paternal X^28^, whereas random XCI is present in human placenta^9^. Due to lack of scRNA-seq data from trophectoderm cells, we were not able to exclude the existence of imprinted XCI in the marmoset.

Male marmoset early blastocysts showed upregulation of *XIST* comparable to that in female cells at the same stage, while in male late blastocysts *XIST* expression was significantly decreased. Interestingly, *XIST* expression has also been detected in rhesus macaques (*Macaca mulatta*) and human male blastocysts^6,7,29^, suggesting that *XIST* expression in preimplantation male embryos is a feature of primates.

We further investigated X upregulation by analysis of the X:A ratio. We showed that male marmoset embryos achieve X:A dosage compensation during the preimplantation development. Accordingly, female marmoset embryos presented an X:A ratio between 1 and 2, suggestive of ongoing XCI in upregulated X chromosomes. In contrast, human embryos achieve X:A dosage compensation only after implantation^11^.

Finally, we investigated the existence of X dampening in the marmoset embryos which should lead to decreased expression levels of biallelically expressed genes during development. Instead, we found no significant differences in expression levels of those genes between the morula and early/late blastocyst stages, refuting the occurrence of dampening.

In conclusion, despite the limited number of female embryos, our work adds to the panorama of XCI in primates, and establishes the marmoset as a model system where, as in humans, X-linked dosage compensation between males and females is achieved by random XCI which starts during preimplantation embryo development.

## Methods

### Transcriptome data

The marmoset (*Callithrix jacchus*) scRNA-seq raw data is available on the ArrayExpress platform (https://www.ebi.ac.uk/arrayexpress/) under accession number E-MTAB-7078. This dataset comprises the transcriptome of 196 cells obtained from 14 preimplantation embryos (from zygote to late blastocyst stage, Table 1).

### Quality filter and mapping

Adapters and low-quality reads were removed using *cutadapt*^30^ v1.18. In addition to removing bases with a *phred* score lower than 20, sequences with poor GC content were also trimmed out. Finally, reads shorter than 30 bp were discarded.

After trimming, TopHat2^31^ v2.1.1 was used to map reads to the marmoset assembly Callithrix jacchus-3.2 and its respective annotation (obtained from NCBI Assembly https://www.ncbi.nlm.nih.gov/assembly/, NCBI *Callithrix jacchus* Annotation Release 102, GCA_000004665.1). SAMTools^32^ v1.9 was used to remove unmapped reads and secondary alignments as well as for sorting. The numbers of reads assigned to each gene were counted with HTSeq using mode *intersection-nonempty*^33^. We used the latest marmoset gene annotation from May 2020 (Callithrix_jacchus_cj1700_1.1).

### Data pre-processing

The marmoset preimplantation embryo dataset contained originally the transcriptome of 196 isolated cells obtained from 14 marmoset preimplantation embryos, from the zygote to the late blastocyst stage. In order to identify cells with low-quality data, a Seurat object was created considering cells with at least 200 detected genes where those genes should be expressed in at least three cells. Through visual analysis we decided to retain only cells with more than 2 × 10^6^ and less than 5.1 × 10^6^ reads mapped to genes (Seurat package version 3.2.2)^34^. After filtering out cells with low-quality data, we retained 124 good quality cells still representing all of the above-mentioned stages.

### Sexing

In order to determine the sex of each embryo, we combined two strategies: (1) counting the number of Y-linked expressed genes, and (2) measuring the expression levels of those genes for each individual embryo. To be considered as an expressed gene, the normalized TPM level should be greater than or equal to one. All Y-linked genes detected in embryos at the zygote and 4-cell stages were removed from the analysis, as the transcriptome at these stages is mainly composed of maternal mRNA deposited during the oogenesis. It is not expected but also not unusual to detect expression of Y-linked genes from female transcriptomes due to the similar regions between the X and Y chromosomes in mammals (mainly the PAR regions). After the filtering steps we retained five protein coding genes located on the non-recombining region of the Y chromosome. Finally, we looked for statistical differences by comparing all embryos at the same stage for both the number of Y-linked genes and their expression level with the unpaired Wilcoxon test.

### Genotyping and variant calling

Genotypes for each embryo were constructed by merging the transcriptome data of all cells from the same embryo and identifying heterozygous positions with the use of VarScan^35^ v2.3.9. Variants were also identified in individual cells with VarScan v2.3.9 and filtered with bcftools^36^ v1.9 in order to keep only biallelic single nucleotide polymorphisms in subsequent analyses. Each cell’s variants were then compared to the respective embryo genotype, a strategy that allows for the identification of differential monoallelic expression in individual cells from the same embryo. Only variants covered by 20 or more reads were considered. GATK^37^ v4.1.0.0 VariantsToTable was used to convert the VCF output from VarScan into a readable format for data analysis.

### Gene calling and allelic expression

Allelic expression was inferred from the allelic frequency of each SNP. According to Borel et al., an allelic frequency of 0–20% or 80–100% of either the reference or alternative allele corresponds to monoallelic expression, while an allelic frequency of 20–80% indicates biallelic expression^38^.

According to the position of each variant, the corresponding gene was identified. Each gene’s allele-specific expression was inferred based on the detected SNPs. When all of the gene’s variants presented monoallelic expression, the gene’s expression was assumed to be monoallelic. The same applies to biallelic expression from the SNPs. If variants from the same gene were not in agreement regarding the allelic expression (mono or biallelic), that gene was discarded from the analysis.

### Allelic expression pattern

The proportion of mono and biallelically expressed genes was calculated as a percentage of the total informative genes in each cell. Informative genes correspond to those genes whose expression can be inferred from their SNPs allele frequency. Total informative genes represent the sum of mono and biallelically expressed genes.

### Gene expression levels

To test the existence of dampening, we extracted the expression levels only for those genes whose expression was found to be biallelic. We then calculated the median expression levels in each cell, taking into consideration the biallelic genes with expression levels greater than 0.1 FPKM (fragments per kilobase of transcript, per million). We employed the same strategy to analyze the expression levels of genes that were found to be expressed monoallelically.

For the evaluation of global expression levels from the X chromosome, we considered all genes with expression levels greater than 0.1 FPKM regardless of being informative (excluding the ones removed from the allele-specific analyses). We then found the median expression levels for each cell and grouped the results according to each embryonic stage.

### X:A ratio

We calculated the median X:A ratio by using all expressed X-linked and autosomal genes (expression level greater than 0.1 FPKM). We also calculated the median X:A ratios according to the method described by Fukuda et al*.*, and only genes with expression levels greater than 0.1 FPKM were considered (excluding the ones removed from the allele-specific analyses)^39^. Using the ‘sample’ function in R, we randomly selected 100 X-linked genes and 100 autosomal genes, for which an X:A ratio was calculated for each cell. This analysis was repeated by bootstrapping with 2000 replications, allowing for the calculation of median X:A ratios (Supplementary Fig. S8).

### Statistics and visualization

Data visualization analyses and statistical tests were performed using R^40^ v3.6.3 and *ggplot2* package^41^ v3.3.2.

## Supplementary Information

Supplementary Figures.

Supplementary Table 1.

## Data Availability

Scripts used in this work can be provided on reasonable request.
