# Supplementary material for: Concurrent X chromosome inactivation and upregulation during non-human primate preimplantation development revealed by single-cell RNA-sequencing
Source: Sci Rep. 2021 May 5;11:9624. doi: 10.1038/s41598-021-89175-7 (PMC8100148; doi:10.1038/s41598-021-89175-7)
Supplement: Supplementary file 1 — Supplementary Figures. [file 41598_2021_89175_MOESM1_ESM.pdf]

SUPPLEMENTARY MATERIAL

Cidral, A.L., Moreira de Mello, J.C., Gribnau J., Pereira, L.V. Concurrent X chromosome inactivation and upregulation during non-human primate preimplantation development revealed by single-cell RNA-sequencing.

SUPPLEMENTARY FIGURE S1: Sexing *C. jacchus* preimplantation embryos. Female embryos: Morula_1, Morula_3, EarlyICM_2, EarlyICM_3, LateICM_4. Male embryos: 8-cell 1 and 2, Morula_2, EarlyICM_1, LateICM_2. (**a**) Number of Y-linked genes expressed in each cell per embryo, where each dot represents a single cell. (**b**) Y-linked gene expression level in each embryo, where each dot represents a single gene. Note that the majority of Y-linked transcripts are detected only in male samples. Embryos at the same stage were compared to each other using an unpaired Wilcoxon test. (**) P-value ≤ 0.01; (***) P-value ≤ 0.001; (****) P-value ≤ 0.0001. Only significant differences are shown. Refer to Supplementary Table S1 for detailed statistical results.

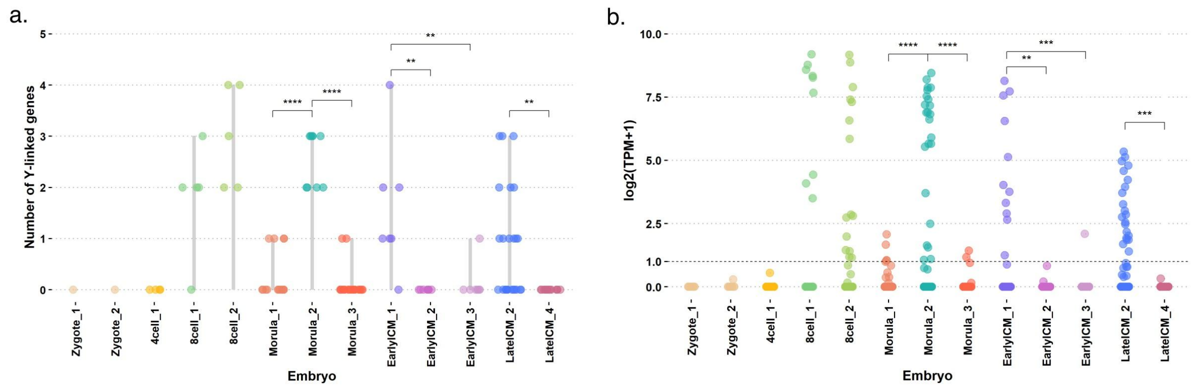

SUPPLEMENTARY FIGURE S2: Allelic expression pattern of X-linked genes in *C. jacchus* by embryonic stage before filtering. Each bar corresponds to a cell. Percentages of mono- and biallelically expressed genes are shown in blue and orange, respectively. (**a**) Zygotes and 4-cell stage embryo, at pre-EGA stage, are expected to present a large proportion of biallelic expressed genes due to maternal mRNA. (**b**) Male embryos showing an unexpected high number of biallelically expressed X-linked genes.

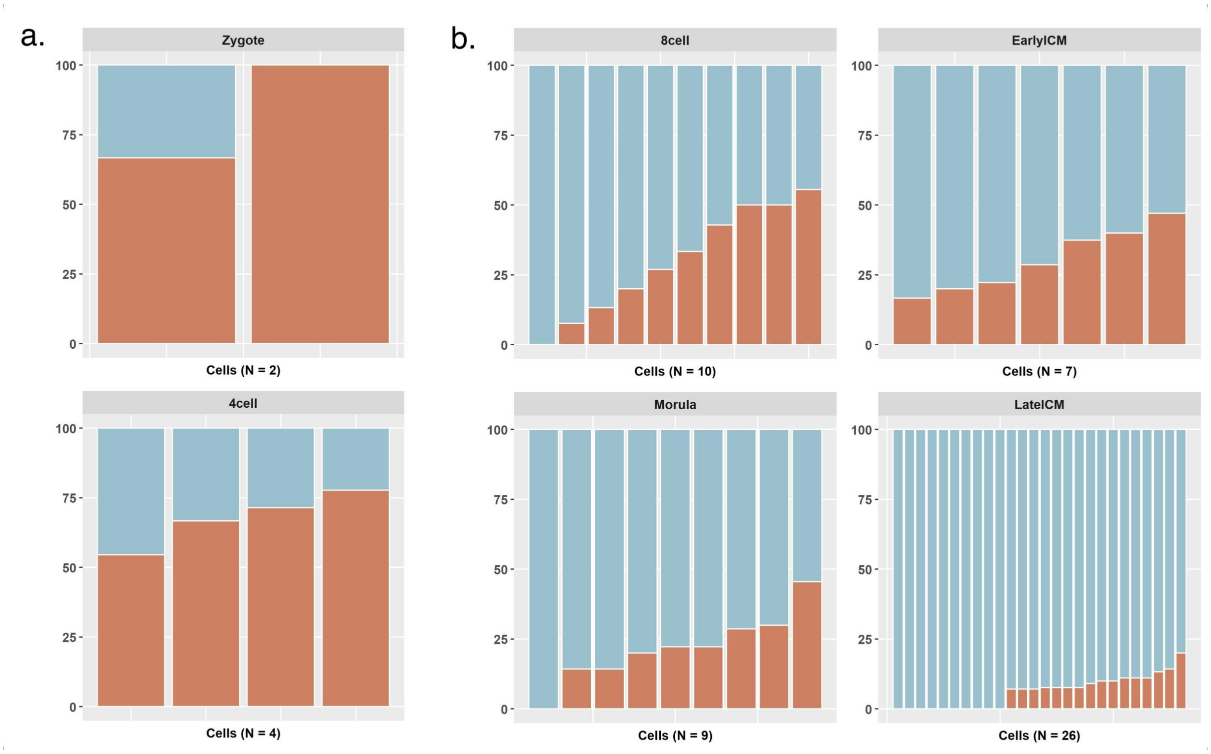

SUPPLEMENTARY FIGURE S3: Allelic expression pattern of X-linked genes in *C. jacchus* by embryonic stage. Each bar corresponds to a cell. Percentages of mono- and biallelically expressed genes are shown in blue and orange, respectively. (**a**) After removing all biallelically expressed X-linked genes detected in male cells, genes homologous to those at the human PAR region and escapees. (**b**) After removing only genes homologous to those at the human PAR region and escapees. (**c**) Autosomal genes: note that there is no striking difference between males and females.

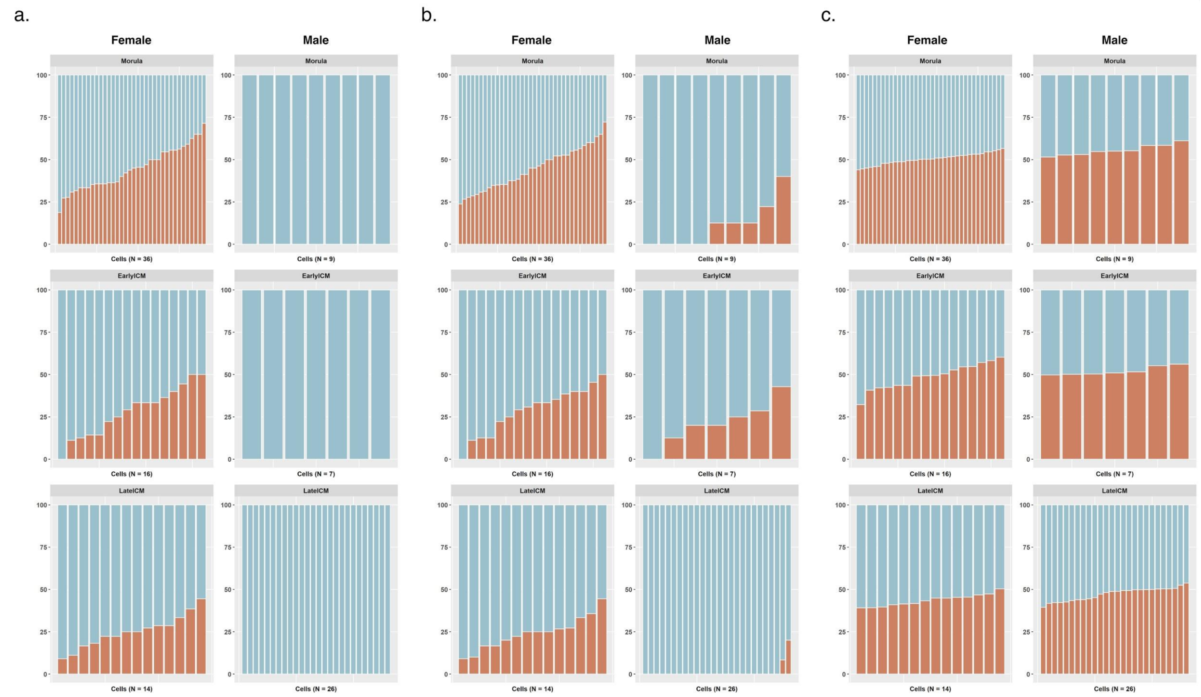

SUPPLEMENTARY FIGURE S4: Allelic expression pattern of X-linked genes after removing only genes homologous to those at the human PAR region and escapees. (**a**) Percentage of monoallelically expressed X-linked genes for each cell according to embryonic stage. Each dot represents a single cell. Top panel: female embryos; bottom panel: male embryos. Pearson's r values (R) are depicted in each panel. Gray area indicates a 95% confidence interval. (**b**) Expression patterns of SNPs along the X-chromosome of female embryos upper panels and male embryos lower panels. The color of each position is assigned according to the ratio of allelic expression based on the detected SNP; blue corresponds to monoallelic expression, while red indicates biallelic expression. Each column corresponds to a single cell. The solid grey lines indicate the telomeres; the three gray lines in grey to the centromere; and the dashed line corresponds to the putative marmoset XIST locus. Top panel: female embryos; bottom panel: male embryos.

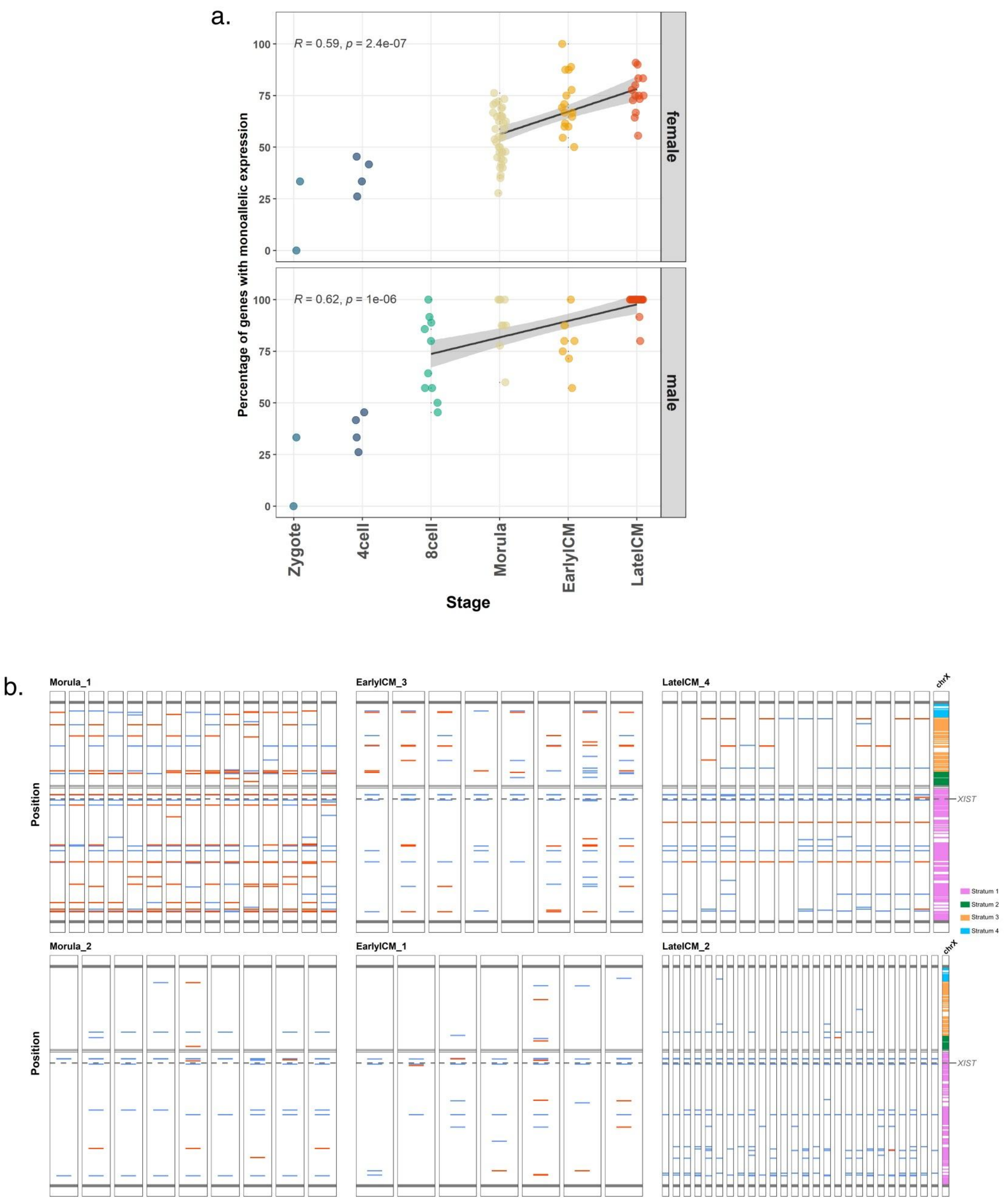

SUPPLEMENTARY FIGURE S5: Allelic expression pattern of genes mapped to chromosome 10. (a) Percentage of monoallelically expressed genes for each cell according to embryonic stage. Each dot represents a single cell. Top panel: female embryos; bottom panel: male embryos. Pearson's r values (R) are depicted in each panel. Gray area indicates a 95% confidence interval. (b) Expression patterns of SNPs along chromosome 10 of female embryos upper panels and male embryos lower panels. The color of each position is assigned according to the ratio of allelic expression based on the detected SNP; blue corresponds to monoallelic expression, while red indicates biallelic expression. Each column corresponds to a single cell. The solid grey lines indicate the telomeres; the three lines in grey to the centromere. Note that there is no striking difference between males and females as well as among different stages. Top panel: female embryos; bottom panel: male embryos.

a.

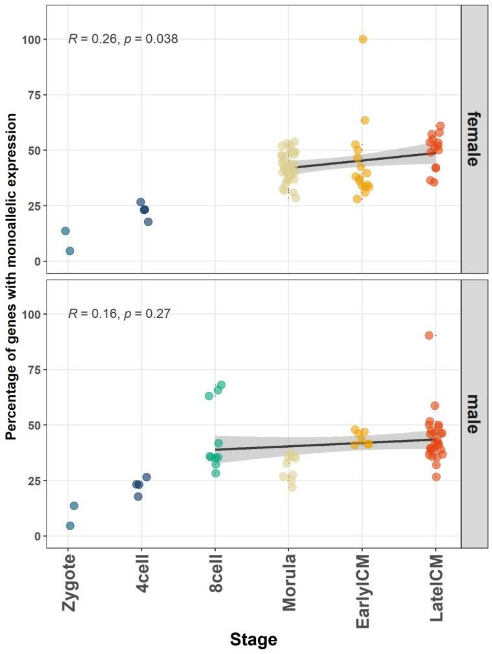

b.

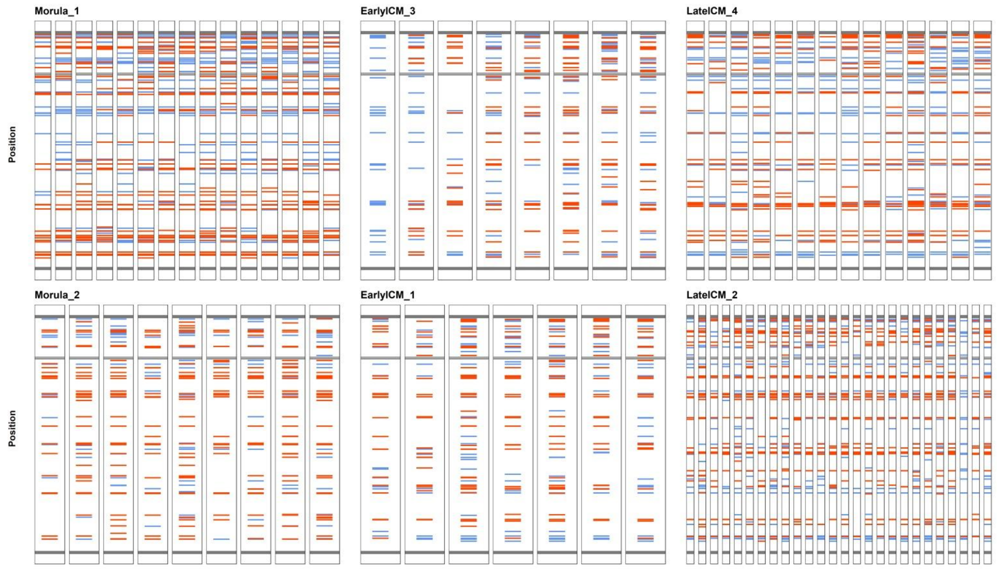

SUPPLEMENTARY FIGURE S6: Tracks of scRNA-seq data showing reads from two cells (cells 96 and 102) of the same early blastocyst with expression of different *XIST* alleles. Cell 96 presented 14 reads covering the relevant SNP position, below the threshold of 20 reads. Image obtained from Integrative Genome Viewer[1].

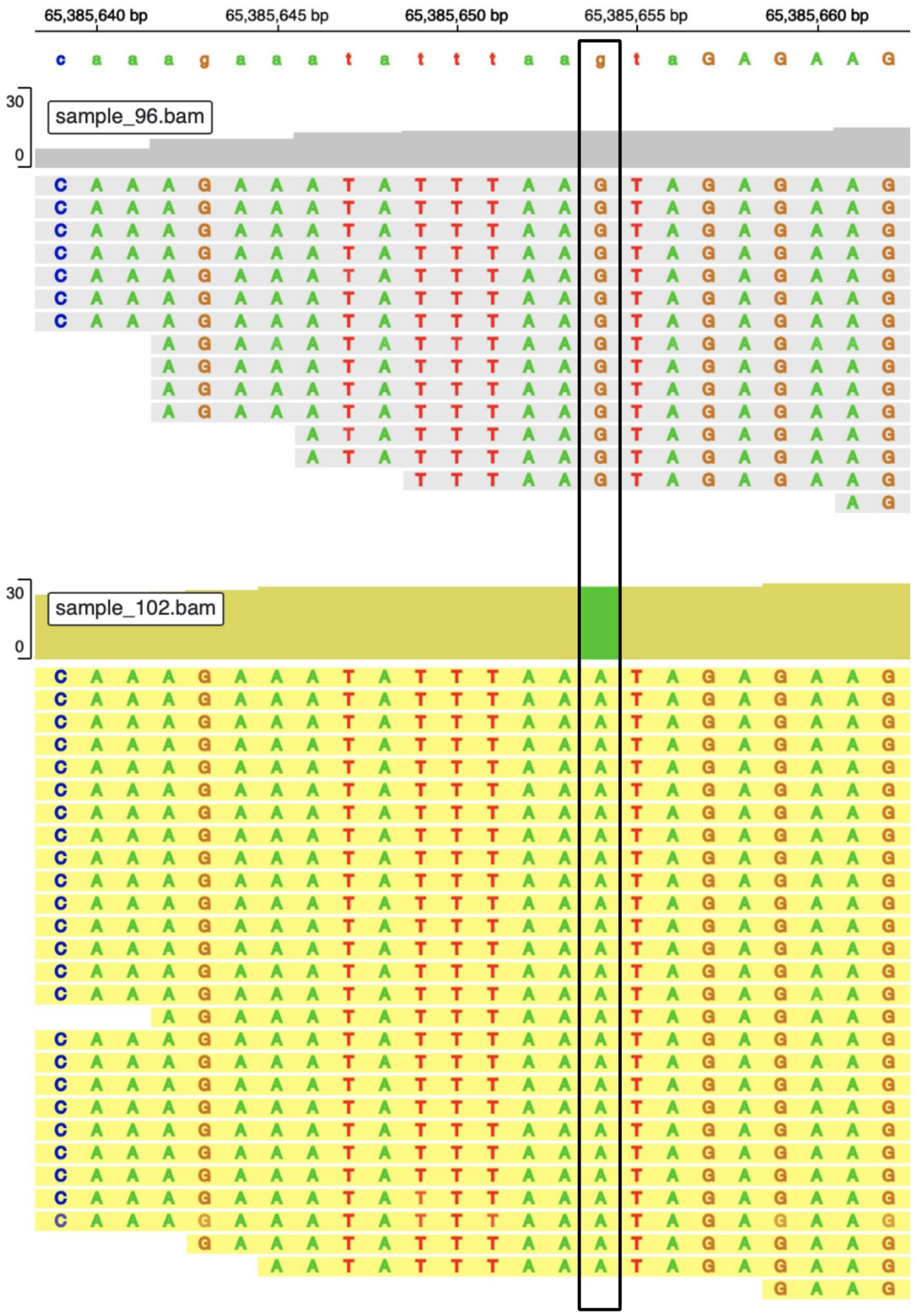

SUPPLEMENTARY FIGURE S7: Expression levels (TPM) of X-linked genes in marmoset embryos. Similar to figure 3 in the main text but removing only genes homologous to those at the human PAR region and escapees. Distribution of the median expression levels of X-linked genes as follows: (**a**) left panel: biallelically expressed; middle panel: monoallelically expressed; right panel: all X-linked expressed genes in each cell of female embryos (biallelic, monoallelic and non-informative). (**b**) Median expression levels of all expressed X-linked genes in each cell of male embryos. Each dot represents a single cell. Stages were compared to each other using an unpaired Wilcoxon test. (*) P-value ≤ 0.05; (**) P-value ≤ 0.01; (***) P-value ≤ 0.001; (****) P-value ≤ 0.0001; (ns) not significant (P-value ≥ 0.05).

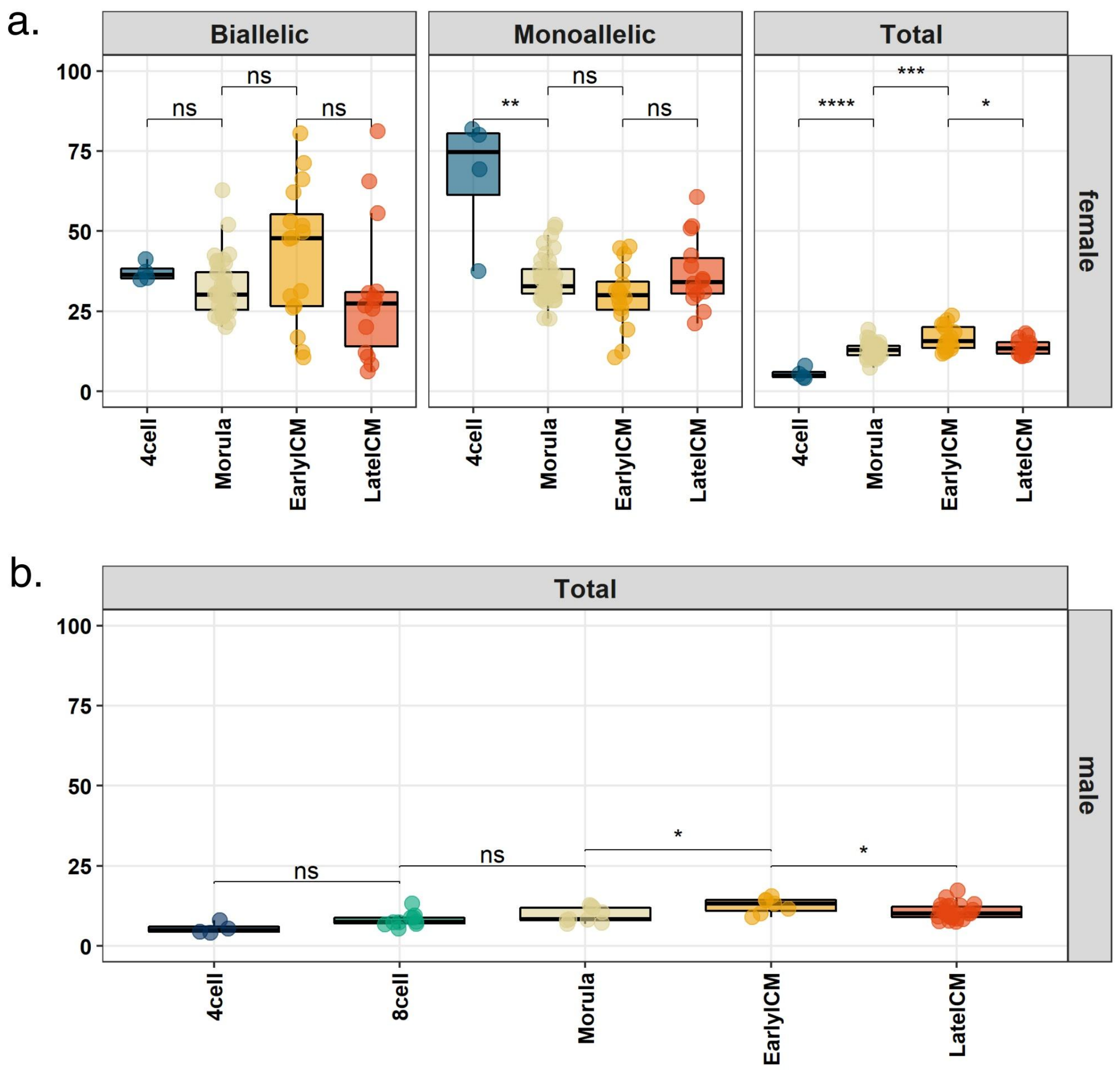

SUPPLEMENTARY FIGURE S8: X-to-autosome dosage compensation using 100 genes randomly selected 2000 times (see Methods). (**a**) X:A ratio distribution calculated per cell of each embryonic stage during marmoset preimplantation development. Unpaired Wilcoxon tests were used to compare males and females at the same developmental stage. (***) P-value ≤ 0.001; (****) P-value ≤ 0.0001. (**b**) same as in (a) with one panel for each sex. Top panel: female embryos; bottom panel: male embryos. Note that, although not sexed, zygotes and 4-cell stage embryos are duplicated in order to maintain panels uniform. Unpaired Wilcoxon tests were used to compare different stages in males and females separately. (ns) not significant (P-value ≥ 0.05).

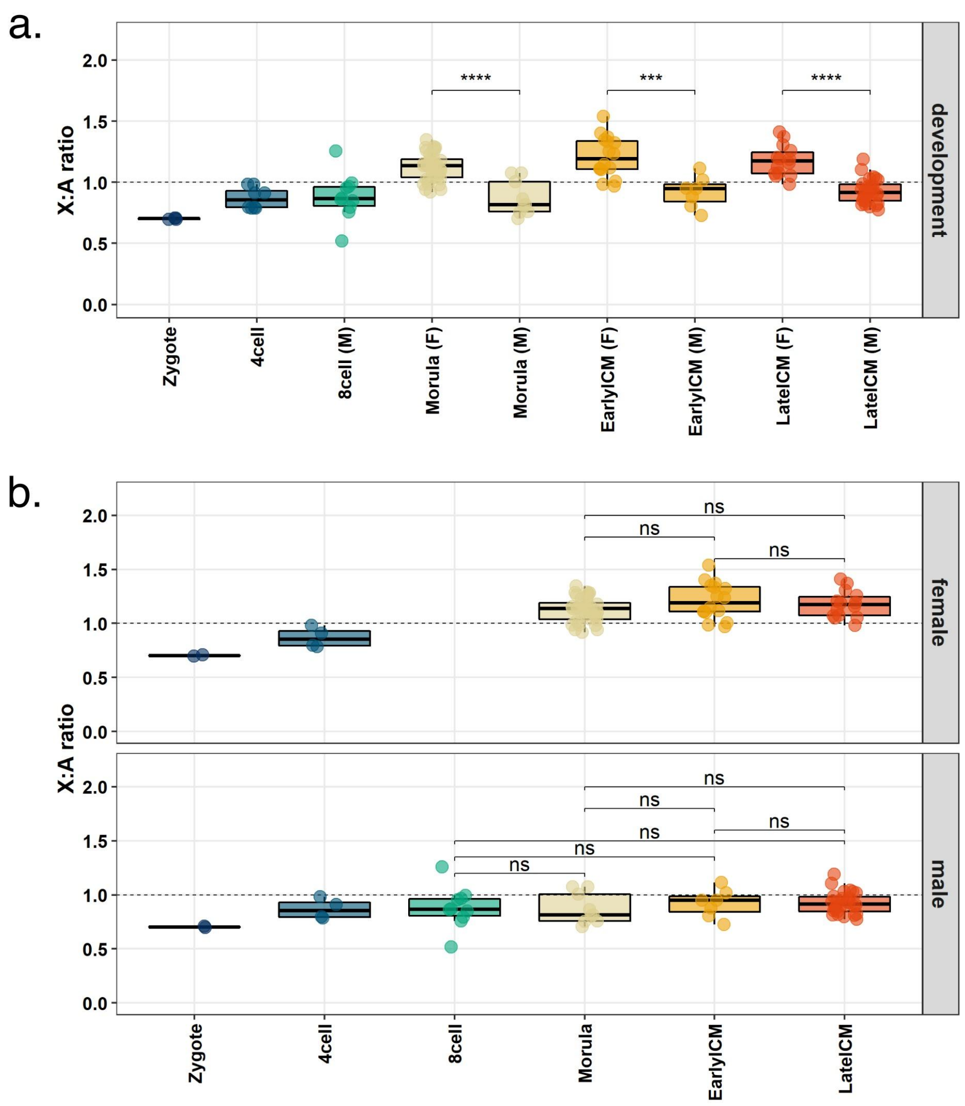

REFERENCES:

1. James T. Robinson, Helga Thorvaldsdóttir, Wendy Winckler, Mitchell Guttman, Eric S. Lander, Gad Getz, Jill P. Mesirov. Integrative Genomics Viewer. Nature Biotechnology 29, 24–26 (2011).
